# Highly Colistin-resistant *Aeromonas jandaei* from a Human Blood Sample

**DOI:** 10.14789/jmj.JMJ22-0047-R

**Published:** 2023-05-20

**Authors:** TOMOKI KOMEDA, SHOVITA SHRESTHA, JATAN B. SHERCHAN, MARI TOHYA, TOMOMI HISHINUMA, JEEVAN B. SHRECHAND, TATSUYA TADA, TERUO KIRIKAE

**Affiliations:** 1 Department of Microbiology, Juntendo University School of Medicine, Tokyo, Japan; 1 Department of Microbiology, Juntendo University School of Medicine, Tokyo, Japan; 2 Department of Microbiology, Institute of Medicine, Tribhuvan University, Kathmandu, Nepal; 2 Department of Microbiology, Institute of Medicine, Tribhuvan University, Kathmandu, Nepal; 3 Department of Clinical Microbiology, Kathmandu University School of Medical Sciences, Dhulikhel, Nepal; 3 Department of Clinical Microbiology, Kathmandu University School of Medical Sciences, Dhulikhel, Nepal

**Keywords:** *Aeromonas jandaei*, colistin resistance, phosphoethanolamine transferases, *mcr*, Gram-negative bacteria

## Abstract

*Aeromonas* species are Gram-negative rods known to cause infections such as gastroenteritis, bacteremia and wound infections. Colistin is one of few treatments for multidrug-resistant Gram-negative bacteria. However, colistin-resistant bacteria carrying the mobilized colistin resistance (*mcr*) gene are a threat in healthcare settings worldwide. In recent years, colistin-resistant *Aeromonas* species have been detected in environmental and clinical samples. We analyzed the genomic characteristics of one highly colistin-resistant *A. jandaei* isolated from a blood sample in Nepal, which harbored four novel *mcr-like* genes on its chromosome. Our study strongly suggests that *A. jandaei* is a reservoir of colistin-resistant genes. Inappropriate use of drugs in medicine and food production should be reduced and continued global surveillance for colistin-resistant bacteria is necessary.

## Taxonomy of the *Aeromonas* genus

The *Aeromonas* genus are Gram-negative, straight, rigid, nonsporeforming rods which belong to the family *Aeromonadaceae*. They are facultatively anaerobic and widely distributed in aquatic environments and food samples. They are frequently isolated from drinking water, wastewater, seawater, livestock, vegetables, seafood and fish^1)^. Although not generally considered marine organisms, they grow naturally in marine systems in contact with freshwater and are found at all salinities except extreme^2)^. The etymology of the word “*Aeromonas*” is that “*Aero*” means “gas(-producing)” and “*monas*” means “*monad*.” The word “monad” is derived from the Greek word “monos,” meaning “one.” Type species of the genus is *A. hydrophila*, and “*hydrophila*” means “water-loving.” Historically, species in this genus were classified in the family *Pseudomonadaceae* or the family Vibrionaceae^3-4)^. After the analysis of 16S ribosomal RNA of the *Ganmamaproteobacteria*, the *Aeromonas* genus became independent from the family *Vibrionaceae* and the family *Aeromonadaceae* was founded in 1992^5)^.

**Figure 1:**
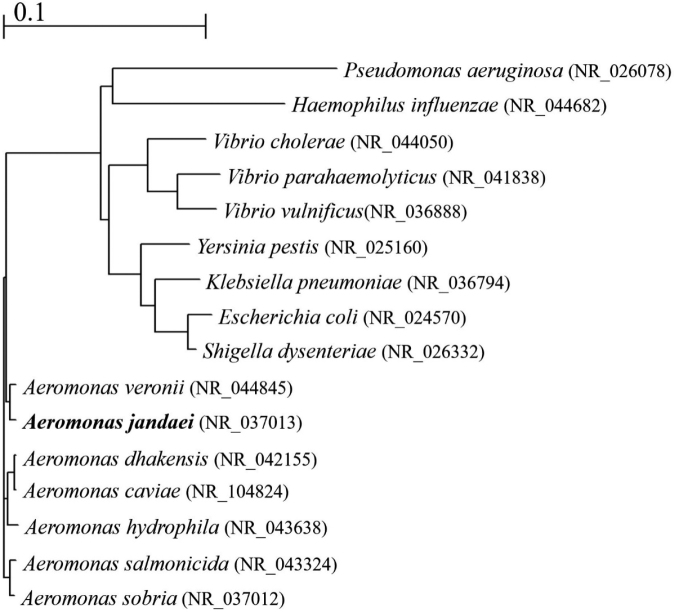
Phylogenetic dendrogram of 16S rRNA of the Gram-negative rods including the family *Aeromonadaceae*. This Figure is created using the CLUSTAL OMEGA program (https://www.ebi.ac.uk/Tools/msa/clustalo/). GenBank accession numbers were indicated in parenthesis.

A Phylogenetic dendrogram of the major type strains of the family *Aeromonadaceae* and clinically important Gram-negative rods is shown (Figure 1).

## *Aeromonas* species as human pathogens

*Aeromonas* species were initially known as fish pathogens. For example, *Aeromonas salmonicida*, discovered in the 19th century, means “salmon- killer.” In the family *Vibrionaceae*, of which *Aeromonas* species were once classified, *Vibrio cholae* and *Vibrio parahaemmolyticus* cause gastrointestinal symptoms, and *Vibrio vulnificus* causes wound infections, especially necrotizing soft tissue infection. Similarly, 19 of 36 *Aeromonas* species are pathogenic to humans, causing a broad spectrum of infections including gastroenteritis, bacteremia, and wound infections^6)^. *A. jandaei*, named after *J. Michael Janda*, was originally isolated from clinical samples including blood, wounds, and stools in the USA in 1991^7)^.

## Emergence of mobilized colistin resistance (MCR)

The emergence of bacterial antimicrobial resistance (AMR) is a widespread problem. According to a report by the UK government, 10 million people could be killed by AMR every year^8)^. In this context, colistin is once again gaining attention as a “last resort” antimicrobial agent against infections with multidrug-resistant Gram-negative bacteria including *Enterobacteriaceae*, *Pseudomonas aeruginosa*, and *Acinetobacter baumannii*, by interacting with lipid A to disrupt the outer membrane of Gram-negative bacteria^9)^.

Colistin was discovered from *Bacillus polymyxa* var. *colistinus*, which was from soil of Fukushima, Japan in 1947 and approved by the U.S. Food and Drug Administration (FDA) in 1959^10)^. However, due to its kidney toxicity, colistin has long been restricted for use in humans. On the other hand, it is used in livestock. As a result, colistin resistant *Escherichia coli* and *Klebsiella pneumoniae* encoding the mobilized colistin resistance gene *mcr-1* on a plasmid were discovered in livestock and humans in China in 2015^11)^. To date, the *mcr* genes have spread across five continents and variants *mcr-1* to *mcr-10* have been found in *E. coli*, *Enterobacter* spp., *Klebsiella* spp., *Proteus* spp., *Salmonella* spp., *Citrobacter* spp., *Pseudomonas* spp., *Acinetobacter* spp., *Kluyvera* spp., *Aeromonas* spp., *Providencia* spp., and *Raulotella* spp.^12-13)^. Transmission of *mcr*- positive bacteria may occur by contact with reservoirs of *mcr*, ingestion of products associated with contaminated animals or plants, and international food trade and travel^13)^. A meta-analysis of colistin- resistant *E. coli* estimated *mcr* prevalence rates of 15.8%, 14.9%, 7.4%, and 4.2% among chickens, pigs, healthy humans, and clinical isolates, respectively^14)^. In Southeast Asia, colistin use as an antibiotic in livestock was so routine that in one rural village survey in Vietnam in 2017, 29 of 36 households tested had colistin-resistant *E. coli* harboring *mcr- 1* or *mcr-3*^15)^.

## Emergence of colistin-resistant *Aeromonas jandaei*

Previous studies have reported that *mcr* related genes were detected in environments, animals, and clinical samples of *Aeromonas* spp. including *A. allosaccharophila*, *A. bivalvium*, *A. caviae*, *A. hydrophila*, *A. jandaei*, *Aeromonas media*, *A. salmonicida* and *A. veronii*^16-20)^. According to the U.S. Department of Agriculture (USDA) study of *mcr* prevalence in 2018, 15 of 5,169 samples were positive for *mcr*: one for *E. coli*, nine for *A. hydrophila*, and five for *A. jandaei*. Of these, all the *Aeromonas* species harbored *mcr-3-like* genes, but only *A. jandaei* harbored *mcr-7-like* gene^16)^. An *A. jandaei* strain isolated from retail fish in China harbored two genes encoding phosphoethanolamine transferase *eptAv3* and *eptAv7* similar to *mcr-3* and *mcr-7*, respectively^21)^. These findings suggest that *Aeromonas* spp. was a reservoir of colistin-resistant Gram-negative bacteria.

## Colistin-resistant clinical isolates of *Aeromonas jandaei* in Nepal

We obtained *A. jandaei* strain JUNP479 (Genbank accession number: AP024466) isolated from a blood sample obtained from an inpatient at Tribhuvan University Teaching Hospital, Nepal, in October 2019^22)^.This strain was resistant to ceftriaxone (MIC 8 mg/L) and intermediate resistant to imipenem (MIC 2 mg/L), but was susceptible to ceftazidime (MIC 0.5 mg/L), meropenem (MIC ≤ 0.25 mg/L), aztreonam (MIC ≤ 0.25 mg/L), amikacin (MIC 4 mg/L), gentamicin (MIC ≤ 0.25 mg/L), ciprofloxacin (MIC ≤ 0.25 mg/L), and tetracycline (MIC 1 mg/L). The MICs of colistin and tigecycline were 128 mg/L and 64 mg/L, respectively^22)^.

## Four novel *mcr-like* genes of *A. jandaei* JUNP479

**Figure 2:**
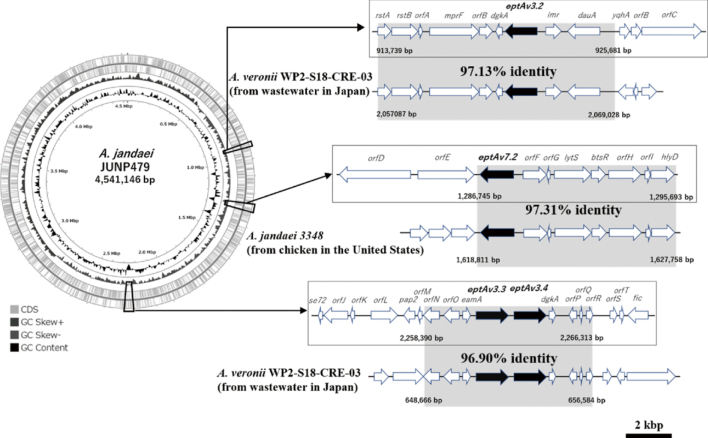
Genomic environments surrounding *eptAv3.2, eptAv3.3, eptAv3.4* and *eptAv7.2* in *Aeromonas jandaei* JUNP479.All four genes encoding phosphoethanolamine transferases were contained in the chromosome. The genomic environments surrounding *eptAv3.2, eptAv3.3* and *eptAv3.4* were similar to those in *Aeromonas veronii* (*A. veronii*) WP2-S18-CRE-03 (accession no. AP021940), whereas that surrounding *eptAv7.2* was similar to that in *Aeromonas jandaei* (*A. jandaei*) 3348 (CP043321)^22)^.

**Figure 3:**
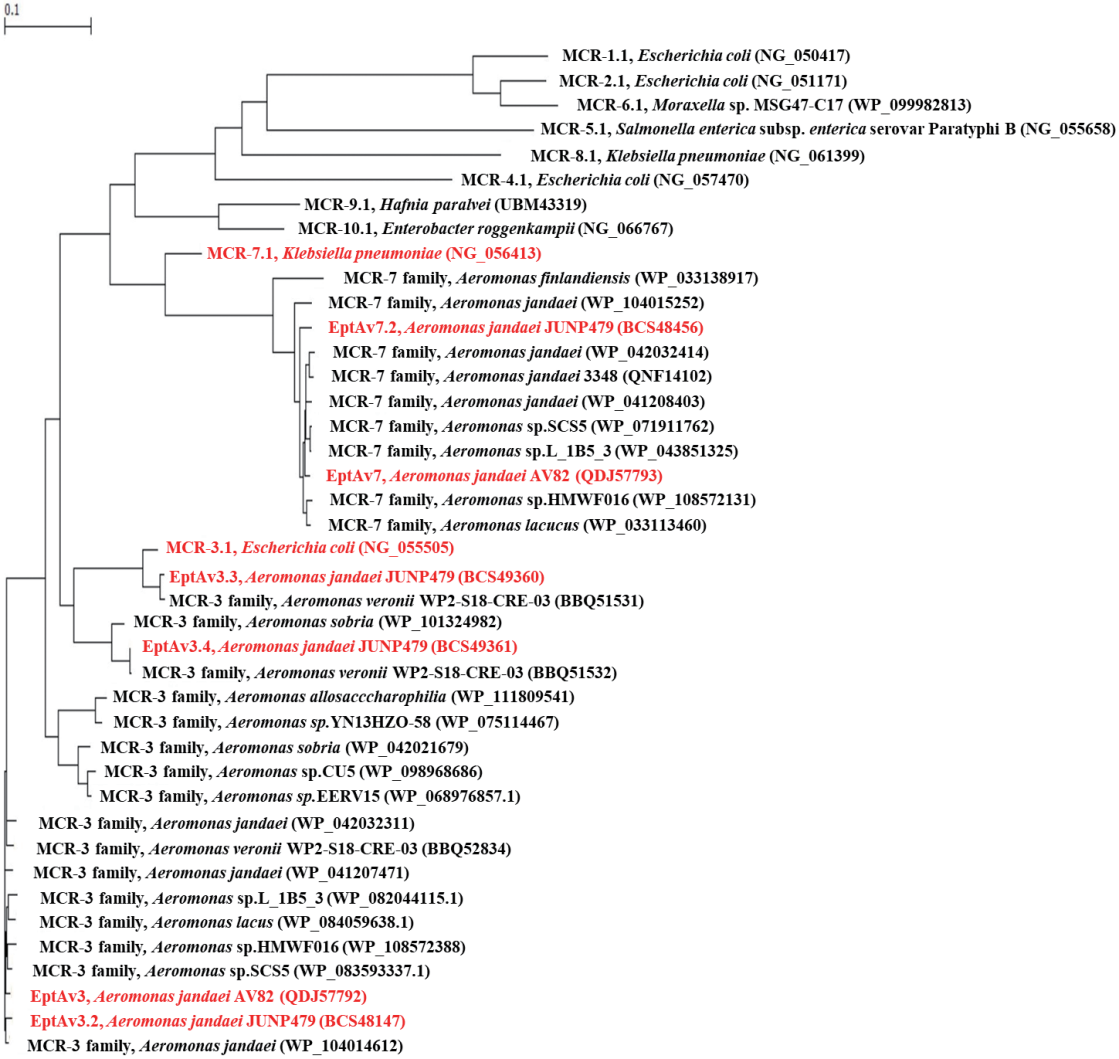
Phylogenetic dendrogram of MCR and EptAv variants. MCR-3.1, MCR-7.1 and EptAv variants were indicated in red. GenBank accession numbers were indicated in parenthesis^22)^.

Whole genome sequencing revealed that *A. jandaei* JUNP479 has a chromosome size of 4,534,922 bp, with 49.93% GC content and a plasmid size of 6,224 bp. This highly colistin-resistant clinical isolate encoded a class C *β*-lactamase *bla*_FOX_, and four novel variants of genes encoding phosphoethanolamine lipid A transferases, designated *eptAv3.2*, *eptAv3.3*, *eptAv3.4*, and *eptAv7.2*. The amino acid sequences of EptAv3.2, EptAv3.3, and EptAv3.4 were 80.7%, 95.7%, and 84.7% identical to that of MCR-3.1, respectively, and 98.1%, 80.4%, and 84.0% identical to that of EptAv3, respectively. The amino acid sequence of EptAv7.2 was 79.9%, and 97.6% identical to the sequences of MCR-7.1 and EptAv7, respectively. All four genes encoding phosphoethanolamine transferases were located on the chromosome, with *eptAv3.3* and *eptAv3.4* forming a tandem structure. The genomic environments surrounding *eptAv3.2*, *eptAv3.3*, and *eptAv3.4* in *A. jandaei* JUNP479 were similar to those in *A. veronii* WP2- S18-CRE-03 (AP021940), whereas that surrounding *eptAv7.2* in *A. jandaei* JUNP479 was similar to that in *A. jandaei* 3348 (CP043321)^22)^ (Figure 2). The mobile elements including transposases and insertion sequences were not detected in the 40,000 bp around the *mcr-like* genes on the chromosome of *A. jandaei* JUNP479. The *mcr-like* genes were introduced into plasmid pHSG398 (Takara Bio, Shiga, Japan) and cloned into *E. coli* DH5α (Takara Bio, Shiga, Japan) and *A. hydrophila* IOMTU903. *E. coli* transformants with *eptAv3.2*, *3.3*, or *3.4*, and in *A. hydrophila* transformants with *eptAv-7.2* increased colistin resistance (Table 1). Real-time PCR showed that the expressions of *eptAv3.2*, *3.3*, and *3.4* in *E. coli* transformants, and the expression of *eptAv7.2* in *A. hydrophila* transformants were increased^22)^. Phylogenetic analysis revealed that MCR-3.1 in *E. coli* (NG_055505) showed close phylogenetic distance to MCR-3 family and EptAv3.3 in *Aeromonas* spp., whereas MCR-7.1 in *Klebsiella pneumoniae* (NG_056413) showed considerable phylogenetic distance from MCR-7 family and EptAv7 variants in *Aeromonas spp*^22)^(Figure 3).

**Table 1:**
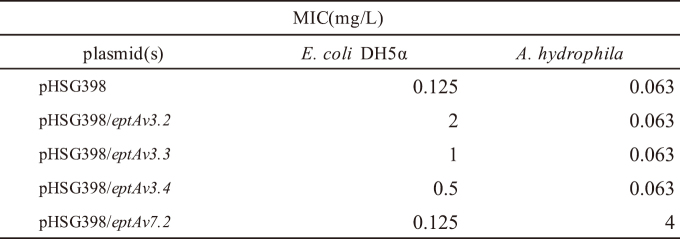
Colistin susceptibility profiles of *E. coli* and *A. hydrophila* transformants^22)^

## Discussion and Conclusion

This is the first report of a highly colistin-resistant *A. jandaei* JUNP479 with four novel genes encoding MCR-like phosphoethanolamine transferases isolated in a medical setting in Nepal. Infections caused by *A. jandaei* are less common than those caused *A. caviae*, *A. veronii*, *A. hydrophila* and *A. dhakensis* in clinical situations^8-9)^. However, when the genomic environments were compared in this study, it turned out that other *Aeromonas* spp. also harbored colistin-resistant genes. Moreover, *mcr-3-like* genes are also commonly found in *Aeromonas* spp. and *E. coli*, suggesting the *mcr* genes may transmit between *Aeromonas* spp. and *Enterobacteriaceae*. On the other hand, *mcr-7-like* genes will mainly spread among *Aeromonas* spp. Our study strongly suggests that *Aeromonas* spp. is a reservoir of colistin-resistant genes, including *mcr-3-like* genes and *mcr-7-like* genes. *Aeromonas* spp. have the possibility to become a threat in healthcare settings, therefore, it is necessary to continue global surveillance of colistin-resistant *Aeromonas* spp. If we humans continue to routinely use colistin in livestock, agriculture, aquaculture, and medicine, we could lose our “last defense” against multidrug- resistant Gram-negative bacteria.

## Funding

This study was supported by grants from Japan Society for the Promotion of Science (Grant Number 21K07031) and Research Program on Emerging and Re-emerging Infectious Diseases from the Japan Agency for Medical Research and Development (Grant Number 21fk0108604h0701).

## Author contributions

TKo summarized the data and drafted the manuscript. SS and JaBS collected samples and analyzed the data. MT and TH performed sequencing and analyzed the data. JeBS, TT and TKi designed the study and supervised the manuscript. All authors read and approved the final manuscript.

## Conflicts of interest statement

No conflict of interest.
